# Functional tissue engineering of ligament healing

**DOI:** 10.1186/1758-2555-2-12

**Published:** 2010-05-21

**Authors:** Shan-Ling Hsu, Rui Liang, Savio LY Woo

**Affiliations:** 1 Musculoskeletal Research Center, Department of Bioengineering, Swanson School of Engineering, University of Pittsburgh, Pittsburgh, PA, USA; 2 Department of Orthopaedic Surgery, Chang Gung Memorial Hospital - Kaohsiung Medical Center, Chang Gung University College of Medicine, Kaohsiung, Taiwan

## Abstract

Ligaments and tendons are dense connective tissues that are important in transmitting forces and facilitate joint articulation in the musculoskeletal system. Their injury frequency is high especially for those that are functional important, like the anterior cruciate ligament (ACL) and medial collateral ligament (MCL) of the knee as well as the glenohumeral ligaments and the rotator cuff tendons of the shoulder. Because the healing responses are different in these ligaments and tendons after injury, the consequences and treatments are tissue- and site-specific. In this review, we will elaborate on the injuries of the knee ligaments as well as using functional tissue engineering (FTE) approaches to improve their healing. Specifically, the ACL of knee has limited capability to heal, and results of non-surgical management of its midsubstance rupture have been poor. Consequently, surgical reconstruction of the ACL is regularly performed to gain knee stability. However, the long-term results are not satisfactory besides the numerous complications accompanied with the surgeries. With the rapid development of FTE, there is a renewed interest in revisiting ACL healing. Approaches such as using growth factors, stem cells and scaffolds have been widely investigated. In this article, the biology of normal and healing ligaments is first reviewed, followed by a discussion on the issues related to the treatment of ACL injuries. Afterwards, current promising FTE methods are presented for the treatment of ligament injuries, including the use of growth factors, gene delivery, and cell therapy with a particular emphasis on the use of ECM bioscaffolds. The challenging areas are listed in the future direction that suggests where collection of energy could be placed in order to restore the injured ligaments and tendons structurally and functionally.

## Introduction

Ligaments and tendons are important structures that are designed to transmit forces and facilitate joint articulation in the musculoskeletal system. As such, these tissues are frequently injured during sports and work related activities. In the case when the anterior cruciate ligament (ACL) and medial collateral ligament (MCL) of the knee as well as the glenohumeral ligaments and the rotator cuff tendons of the shoulder are torn, the respective joints can become functionally disabled while the soft tissue in and around the joints including the cartilage, menisci, and others can be predisposed to damage. In severe cases, ligament and tendon injuries can bring on the early symptoms of osteoarthritis.

The healing responses following injuries to different ligaments and the consequences can vary greatly. The ACL of knee has limited capability to heal, and the results of non-surgical management of its midsubstance rupture have been poor[1,2]. Consequently, surgical reconstruction of the ACL using tissue autografts, such as the bone-patellar tendon-bone (BPTB) or hamstrings tendon (HTs), and soft tissue allografts is regularly performed to gain knee stability. However, there are complications coming with these reconstruction surgeries that include the donor site morbidity, extensor deficit of the knee, degeneration of tissue replacement graft, hamstring muscle weakness, bone tunnel enlargement and other side effects[3-12]. In spite of significant efforts being made to improve the surgical procedures for ACL reconstruction during the last twenty years, many patients still develop osteoarthritis early in the long term[13,14].

Extra-articular ligaments such as the MCL of the knee have a high propensity for healing without surgical management[15-20]. Their structural properties based on tensile testing of the femur-MCL-tibia complex (FMTC), can be restored within weeks, and as a result, patients can return to work and sports quickly with functional treatment using splints or braces. Nevertheless, laboratory studies has discovered that the mechanical properties, histomorphological appearance, and biochemical composition of these healed MCL remain poor when compared to those of the normal MCL[18,21-25]. With the availability of functional tissue engineering (FTE) and the promising use of growth factors, stem cells, and bioscaffolds, research work to improve the tissue quality has been done, especially by means of good animal models such as the rabbit, dog and goat[26]. Much has been learnt about the healing process as well as the potential for extending the novel methods to the healing of other ligaments and tendons including the ACL. Consequently, there is a renewed interest in revisiting ACL healing in order to avoid some of the complications resulted from surgical reconstruction.

In this article, we will first briefly review the biology of normal and healing ligaments and tendons, and then focus on the issues related to the treatment of ACL injuries. Afterwards, we move on to the presentation of promising FTE methods for the treatment of ligament injuries, including the use of growth factors, gene delivery, and cell therapy, but a particular emphasis will be placed on the use of ECM bioscaffolds. To conclude, we will outline some challenging areas and suggest where we should put our energy in order to closely restore the structure and function of injured ligaments and tendons to their pre-injury levels.

## Normal and Healing Ligaments and Tendons

**Figure 1:**
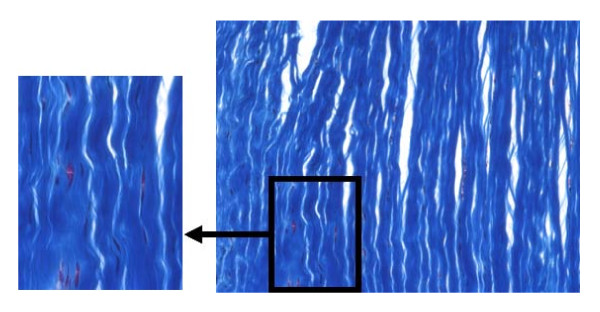
**Histological image of rabbit medial collateral ligament showing highly organized collagen fibers and the spindle shaped fibroblasts (Masson's Trichrome staining at a magnification of 200 ×)**.

Ligaments and tendons are dense connective tissue that connect bone to bone and bone to muscle, respectively. These tissues are relatively hypocellular, as well as hypovascular[27-30]. Collagen fibers are the primary matrix structure, and approximately 70% to 80% of the dry weight of normal tendon or ligament is composed of type I collagen, which is primarily responsible for the stiffness and strength of these tissues. The collagen fibrils that are subunits of collagen fibers are surrounded by extrafibrillar matrix, such as water (65% to 70% of the total weight), elastin (5% to 7% of the dry weight), proteoglycans, and glycolipids[31,32]. Fibroblasts are the predominant cell type and are arranged in rows between bundles of parallel arranged collagen fibrils (Fig. 1). There are also minor types of collagen, including types III, V, X, XI, and XII[33-36]. Type III collagen is responsible for ligament and tendon repair [35] whereas type V collagen is believed to exist in association with type I collagen to regulate the collagen fibril diameter[37,38]. Other collagens such as types XII and XIV, called fibril-associated collagens with interrupted triple helices (FACITs), are localized to the surface of the fibrils[34]. Type XII collagen is thought to provide specific bridges between fibrils and other matrix components, such as decorin and fibromodulin[36] while type XIV collagen is involved in linear fibril growth[39]. Other molecules involved in collagen fibril assembly are a group of small leucine-rich proteoglycans (SLRPs), such as decorin, lumican, biglycan, and fibromodulin[37,40-44]. On the other hand, even though the morphological appearances of ligaments and tendons are similar to each other, there are substantial and important differences in terms of their biochemistry, hence their biomechanical properties[30,45-47].

Generally, ligaments and tendons are metabolically active with incessant cell renewal and matrix turnover albeit at a relatively slow rate[47]. Therefore, after injury, ligaments and tendons heal at a slower rate than most other soft tissue because of their hypovasculariy as well as hypocellularity. Further, their environment would have profound effects on their healing capabilities. For extra-articular ligaments, such as the MCL, the healing is spontaneous and classical. It can be divided into 4 overlapping phases: Phase I is featured by initial bleeding and blood filled into the gap with hemostasis during the initial 72 hours. A hematoma is developed to bridge the torn ends. This area is then infiltrated by inflammatory cells including monocytes, leukocytes, and macrophages that secrete cytokines and growth factors to start the healing process. Phase II, the cellular proliferation phase, is featured by inflammation reaction and granulation tissue formation with the arrival of fibroblasts that slowly populate the injured area and synthesize type III collagen and, to a lesser extent, type I collagen. Phase III has cell proliferation and matrix deposition forming a vascular neo-ligament, while phase IV is featured by the organization of collagenous tissue to be arranged along the functional axis of the ligament as well as synthesis of higher proportion of type I collagen and then long-term remodeling[21].

Investigators have discovered that various cytokines are produced by the infiltrating cells. These endogenous growth factors such as platelet-derived growth factor (PDGF) and transforming growth factor beta (TGF-β) are present in high concentrations during Phase I and II. Studies found that after the initial surge, the level of growth factors started to decrease to baseline level from 2 to 3 weeks of healing[48,49]. Such temporal growth factor responses at the initial phases of ligament healing are critical for the filling of tissue defect with neo-tissue and thereafter the restoration of function.

Knowledge on the mechanisms of ligamentous tissue healing has been accruing rapidly through intense studies, which will no doubt benefit the treatment of injured ligaments through properly designed functional tissue engineering approaches.

## Issues Relating to Healing of the Anterior Cruciate Ligament (ACL)

In the case of the ACL, however, the manner of its healing is entirely different from those described above. Following injury, the thin synovial sheath of ACL is disrupted, and blood dissipates in the synovial fluid, making the formation of a localized hematoma difficult. With such a lack of supply of cytokines and growth factors and a low supply of reparative cells at the injury site, the ability for a torn ACL to heal becomes limited[50-52]. In addition, its torn ends retract significantly because of the high residual strain existed in the intact ACL, making the bridging of the gap even more difficult[52]. Biologically, it is also found that the properties of ACL fibroblasts are different from those derived from other ligaments. They have comparatively low mobility, low proliferation and metabolic activities as well as low matrix production tendencies[53-55]. The cells actually further exhibit higher matrix metalloproteinases (MMPs) activities and poor adhesive strength[56,57]. With all these factors added to the local environmental constraints, the intra-articular ACL rupture, especially at midsubstance, failed to heal on its own.

Clinically, primary repair of ACL using sutures began with A.W. Mayo in 1903 and then followed by O'Donoghue, Feagin, and many others[58-61]. Overall, the results had not been encouraging as they were not different from conservative treatment[59,62-67]. As much as 70% of the patients had knee instability[59,65,67]. Therefore, ACL reconstruction using autografts and allografts has become popular for a treatment. It is estimated that over 100,000 ACL reconstructions are performed in the United States annually with the majority of which using either hamstrings or bone-patellar tendon-bone autografts[68-73]. Although the use of the latter offers the advantage of direct bone to bone fixation for better initial knee stability, the associated problems such as donor site morbidity, knee pain, extensor deficit, and other side effects have led many surgeons to use the hamstrings autograft[10,66,74-80]. Nevertheless, there are problems associated with bone-soft tissue healing, less knee stability, tunnel enlargement, graft motion in the bone tunnels, etc.[9-11,14]. In either case, many patients had good knee stability and after a period of rehabilitation following surgery, they could return to work or sports. However, in the long term, 20-25% of the patients showed less than satisfactory results with some progressing to knee osteoarthritis[81-87].

## Functional Tissue Engineering for ACL Healing

More recently, efforts have begun to focus on alternative approaches that can avoid the problems that associated with ACL reconstruction. A healed ACL has many advantages including the preservation of its native insertion sites as well as its proprioceptive function. Clinical techniques like the 'healing response' by making microfracture holes in the femur close to the ACL insertion was pioneered by Steadman. It aims to introduce blood clot to the injured ACL encouraging hematoma formation and bring in more reparative cells to heal the torn ACL[51,88,89]. For patients over 40 years of age that have proximal ACL tears, this procedure has successful results[88]. On the other hand, there are also experimental evidences showing that a transected ACL might heal with exogenous aids, such as the supplementation of growth factors or use of a scaffold[90-94]. It has been shown that the ACL cells can proliferate and make matrix following FTE treatment[95,96].

FTE is a new field that combines morphology, molecular biology, biochemistry, biomechanics, and other areas. For ligaments, it is particularly important to consider their functional roles in the design and development of novel FTE approaches including the use of growth factors, gene transfer/gene therapy, cell therapy, and extracellular matrix bioscaffolds. Specifically, besides the encouragement for cell proliferation and matrix production, the unique characteristics of the dense regular connective tissue, the natural anatomical insertions to the bones as well as the structural and mechanical properties that are critical for the function of ligaments to sustain and transfer loads, should also be the targets of an optimal FTE treatment. Previous works had reported the use of hyaluronic acid (HA), basic fibroblast growth factor (bFGF), collagen-platelet rich plasma (C-PRP) as well as stem cells to heal the central ACL defects[90-94], and all have shown an increased vascularization, increased tissue formation as well as improvements in some of the biomechanical properties. The following is a brief review of more recent available approaches aiding in the healing of ACL in the laboratory. These methods are the major biological augmentations used in the field of tissue engineering.

### Growth factors

Due to their important physical functions in the regulation of cell responses to injury, the use of growth factors can be advantageous to heal injured ligaments. In the literature, different growth factors such as FGF, TGF-β, PDGF, epidermal growth factor (EGF), insulin-like growth factor (IGF), growth and differentiation factor (GDF) and nerve growth factor (NGF) have been shown to improve vascularization and new tissue formation that resulted in improved structural properties of ligament-bone complex[97-101]. These growth factors also exhibited positive effects on improving ACL healing. In an ACL central defect model in dogs, the bFGF pallets caused healing tissue formation with increased vascularity at early stage compared to little or no tissue formation in the control[94]. In addition, the application of PRP, which contains increased presence of various growth factors, was also reported. It was found that the collagen-PRP complex could significantly increase the tissue formation of an ACL central defect in a canine model and enhance the structural properties of the femur-ACL-tibia complex (FATC) of a completely transected ACL after primary repair in a porcine model[92,102,103].

The potential of synergistic effects of two or more growth factors has also been explored. A combination of PDGF-BB/TGF-β1 did not enhance the structural properties of the healing FMTC compared to the use of PDGF-BB alone[104]. Clearly, the healing process of ligaments is much more complex than simply supplementing certain growth factors. Considering the milieu around the healing tissue differs in location and changes with time, strategies of treatment could be more specific. Further, growth factors have short half-lives, which have limited their efficacy. Therefore, safe and reproducible delivery systems that would allow sustained delivery of growth factors to the injury site need to be vigorously investigated[105-107]. Potential of using synthetic PLGA microspheres, fibrin-heparin delivery system, and metallic porous materials and so on as well as refinement of these systems are being investigated[108].

### Gene transfer/Therapy

Gene transfer using carriers including both retroviral and adenoviral vectors as well as liposomes have been used to induce DNA fragments into healing ligaments by promoting or depressing the expression of certain genes[109]. An *in situ *gene transfer of TGF-β1 using an adenoviral vector in a collagen hydrogel placed between the stumps of a ruptured ACL resulted in an increase in the cellularity and the deposition of type III collagen[110]. Similarly, transfer of IGF-1 cDNA by using an adenovirus vector led to the synthesis and deposition of increased amounts of types I and III collagen, elastin, tenascin, and vimentin in the same model[111]; thus confirming the potential of using vector-laden hydrogels for the *in situ *delivery of genes to damaged ligaments for potential biological repair of the ACL.

### Cell therapy

Mesenchymal progenitor cells (MPCs) and mesenchymal stem cells (MSCs) have shown tremendous potential in tissue engineering[112,113]. MSCs isolated from a variety of adult tissues including the bone marrow (BM) have the capacity to differentiate into different cell types and therefore are attractive to be used as a potential therapeutic tool for tissue repair. In our research center, it was found that MSCs implanted in the injured rat MCL differentiated into fibroblasts[114]. Further, when an MSC-seeded implant was delivered to an Achilles tendon with 1cm gap injury, the healing tissue was grown with a significantly larger cross-sectional area, and the collagen fibers appeared to be better aligned than those in the controls[115]. Similarly, an autologous MSC collagen graft could accelerate the healing as well as improve the quality of healing tissue of patellar tendon in rabbits[116]. Knowing these positive findings, an intra-articular injection of bone marrow derived mysenchymal cells in a rat model with partially transected ACL was done and the formation of healing tissue was found. Consequently, the ultimate failure load of FATC was increased when compared to non-treated control[117]. These results are encouraging because the MSCs have the potential to serve as a vehicle for delivering therapeutic molecules as well as directly enhance the healing of ligaments.

Although it is an appealing property that the MPC/MSCs have the potential to differentiate into many kinds of cell types, how to differentiate these multi-pluropotent cells into a desired specific cell type are still under investigation. Thus, research in the field presents new challenging opportunities in developing novel techniques for optimizing the stem cell system as well as their application in the regeneration of ACL.

### Extracellular matrix bioscaffolds

Bioscaffolds derived from extracellular matrix (ECM), such as the porcine small intestinal submucosa (SIS) and urinary bladder membrane (UBM), have been found to support tissue regeneration and repair of ligaments and tendons[118-129]. SIS is mainly composed of collagen (90% of dry weight) and contains cytokines and growth factors such as FGF and TGF-β[130,131]. It is a resorbable bioscaffold that can provide a collagenous structure for the healing cells to reside as well as hold nutrients necessary for healing[118].

**Figure 2:**
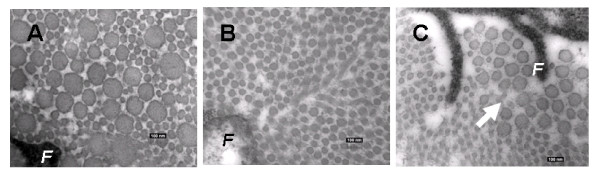
**Transmission electron microscopy images of cross sectional view of collagen fibrils in (A) Normal MCL; (B) Healing MCL at 6 weeks; and (C) SIS-treated healing MCL at 6 weeks.** F indicates fibroblasts. Arrow points to the large newly formed collagen fibrils.

We have applied the SIS bioscaffold to treat a central third defect of patellar tendon in a rabbit model, which is commonly the donor site of autografts for ACL reconstruction. It was found that the bioscaffold could encourage neo-tissue formation in the defect and consequently, the structural properties of the bone-patellar tendon-bone construct were significantly improved[132]. Further, with a single layer of SIS applied to a 6mm gap injury of the rabbit MCL, the quality of the healing tissue was significantly improved. The morphology showed aligned collagen fibers, while the gene expressions of the fibrillogenesis-related molecules such as collagen V and some SLRPs were down-regulated with concomitant increases in the collagen fibril diameters (Fig. 2). Correspondingly, the tangent modulus and the stress at failure of the healing MCL were increased by about 50%[123-126].

**Figure 3:**
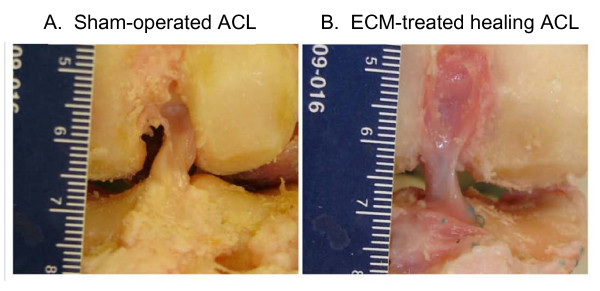
**Gross morphology of (A) Sham-operated ACL; and (B) ECM-treated healing ACL at 12 weeks (permission requested from Woo et al**. [133]).

With these successes, we have used the SIS bioscaffolds for ACL healing. Using a goat stifle joint as a model, we combined the SIS bioscaffold with SIS hydrogel to heal a transected ACL following primary repair[133]. After 12 weeks, the gap was filled with continuity of neo-tissue formation with a similar cross-sectional area and shape as the sham-operated ACL. The neo-tissues were slightly reddish in color and less opaque than the sham-operated control ACLs which indicated that the fibers in the neo-tissue was still not as dense (Fig. 3). Histologically, the collagen fibers were aligned with spindle shaped fibroblasts at 12 weeks. Functional measurements on knee kinematics and in-situ forces were done using a novel robotic/universal force-moment sensor (UFS) testing system developed in our research center[25,134,135]. When an external 67 N anterior-posterior (A-P) tibial load was applied to the stifle joint at flexion angles of knee 30°, 60°, and 90°, the resulting A-P joint instability in the ECM-treated group were significantly reduced to 63%, 49%, and 47% of those for the ACL-deficient joints, respectively. Meanwhile, in-situ forces of the neo-ACL were similar to those of the intact ACL. Together, these data suggest that the ECM treated healing ACLs could contribute positively to knee function. Uniaxial testing of the FATC also showed that the tensile stiffness of the ECM-treated ACL reached 42% of the normal ACL at 12 weeks post-surgery, which was comparable to the results of ACL reconstruction. These findings indicate that the application of ECM bioscaffolds plus ECM hydrogel should have the potential to be a good candidate tool for ACL healing.

## Future directions

Research to enhance ligamentous tissue healing and regeneration has reached an exciting time as new developments on both biological and biomechanical augmentation can be used to improve their outcome. With functional tissue engineering, the ECM bioscaffolds could be further improved via mechanical stimuli and cell seeding to alter their ultrastructure to be closer to that of the highly aligned collagen fiber network of native ligaments [136-139]. Another area for future studies will involve the use of the ECM bioscaffolds derived from genetically modified pigs, such as those with the galactose α1,3-galactose (αGal) deficiency, to reduce hyperacute rejection of the xenograft in humans[140]. With the reduction or elimination of the immunogenicity from the ECM bioscaffolds, its usage will be more widely acceptable [141-144].

Studies should also be done to control the release of growth factor. New delivery system will be needed such that the sustained release of growth factors could stimulate the healing process over time in order to mimic the expression of growth factors *in vivo *that last long time after tissue injury.

Finally, there is another class of scaffolds that will be available in the field, i.e., biodegradable metallic materials such as porous magnesium or magnesium oxide, that have the potential to facilitate ligament and tendon healing and regeneration[145-147]. The advantages of these "smart" scaffolds include their initial stiffness and controllable degradation rate as they are replaced by the neo-tissue formation. It is also possible to protein-coat these metals for better tissue integration and control release of growth factors and cytokines to sustain tissue healing as well as to guide tissue regeneration.

## Conclusions

Clearly, much work remains but there are exciting possibilities. All will require much interdisciplinary and multidisciplinary research. We believe that when biologists, biochemists, clinicians, and bioengineers are teaming together with other experts, it will be possible to make positive advances on ligament and tendon regeneration. In the end, more complete recovery of these tissues will allow patients to resume their daily activities as well as sports.

## Competing interests

The authors declare that they have no competing interests.

## Authors' contributions

SH, RL and SLYW participated in the review design, literature search, coordination and drafting the manuscript. All authors read and approved the final manuscript.
